# From knowledge to action: strengthening cancer prevention knowledge in schools among adolescents in Germany

**DOI:** 10.1186/s12889-026-26442-0

**Published:** 2026-02-03

**Authors:** Nicole Erickson, Irina Rupp, Hansjörg Baurecht, Christine Welker, Friederike Mumm, Claudia Mück, Nicole Jost, Sabine Verena Kesting, Alessandra Holzem, Lucie Heinzerling, Hana Algül, Volker Heinemann, Theres Fey, Julian Walter Holch

**Affiliations:** 1 Comprehensive Cancer Center (CCC Munich LMU), Ludwig Maximilian University (LMU) Hospital Munich, Pettenkoferstraße 8a, Munich, 80336 Germany; 2 Department of Epidemiology and Preventive Medicine, University of Regensburg, Regensburg, Germany; 3 Department of Medicine III, Ludwig Maximilian University (LMU) Hospital Munich, Munich, Germany; 4 Krebsberatungsstelle Lebensmut E.V. Am CCC München LMU, Munich, Germany; 5 Technical University of Munich, Munich, Germany; 6 TUM School of Medicine and Health, Department of Pediatrics, German Center for Child and Adolescent Health (DZKJ), Partner Site Munich, Munich, Germany; 7 Department of Dermatology and Allergology, Ludwig Maximilian University (LMU) Hospital Munich, Munich, Germany; 8 Comprehensive Cancer Center München (CCC TUM), University Hospital Rechts Der Isar, Technical University Munich, Munich, Germany; 9 Department of Internal Medicine II, TUM Hospital Klinikum Rechts Der Isar, Technical University Munich, Munich, Germany; 10 Bavarian Cancer Research Center (BZKF), Ludwig Maximilian University (LMU) Hospital Munich, Munich, Germany

**Keywords:** Cancer Prevention, Adolescents, European Code against Cancer, Knowledge, Health Literacy, School-based Program, Public Health

## Abstract

**Background:**

This prospective interventional study assessed adolescents’ baseline knowledge of the 12 European Code Against Cancer (ECAC) recommendations and evaluated the impact of a school-based multimedia enhanced intervention on knowledge improvement and retention.

**Methods:**

The intervention comprised six 45-min multimedia lessons promoting knowledge of all 12 ECAC recommendations in German secondary schools. Materials were developed and refined by an interdisciplinary expert panel and underwent pilot testing before implementation. An anonymous single-recall-based, open-ended questionnaire assessed baseline knowledge (t0) and mean knowledge gains immediately post-intervention (t1) and at three months follow-up (t2). Descriptive statistics were computed, with quantitative variables summarized by mean and standard deviation, and qualitative variables by absolute and relative frequencies.

**Results:**

A total of 923 pupils participated (51.8% female; 39.0% male; 9.2% diverse/undeclared), with 923 completing baseline (t0), 873 post-intervention (t1), and 779 the follow-up assessments (t2). Notable knowledge gaps regarding the ECAC were present at baseline. Mean (M) knowledge scores increased significantly across all three assessment time points with post-intervention scores (t1, M = 7.63 (Standard deviation (SD) = 2.67); *p* < 0.0001) significantly higher than at baseline (t0, M = 4.11 (SD = 1.84)). Largest average improvements post intervention (t0-t1) were observed for breastfeeding importance (+ 50%; 1.20% (t0), 51.2% (t1), *p* < 0.001), vaccination participation (+ 49.6%; 13.9% (t0), 63.5%(t1), *p* < 0.001) and regular physical activity (+ 39.4%, *p* < 0.001).The most sustainable recommendation improvements [calculated by (t2-t0)/(t1-t0)] were observed for alcohol abstinence (0.76); healthy dietary pattern (0.72), and physical activity (0.69) respectively. Conclusions: Multimedia enhanced school-based interventions incorporating the ECAC recommendations effectively increase cancer prevention knowledge among adolescents and knowledge retention after three months. While knowledge retention trends indicated a need for reinforcement, our results demonstrate the effectiveness of early, targeted interventions to address baseline knowledge gaps and provide insights that could potentially shape future interventions.

**Conclusions:**

Multimedia enhanced school-based interventions incorporating the ECAC recommendations effectively increase cancer prevention knowledge among adolescents and knowledge retention after three months. While knowledge retention trends indicated a need for reinforcement, our results demonstrate the effectiveness of early, targeted interventions to address baseline knowledge gaps and provide insights that could potentially shape future interventions.

**Supplementary Information:**

The online version contains supplementary material available at 10.1186/s12889-026-26442-0.

## Highlights

• Knowledge gaps exist among German adolescents about cancer prevention risk reduction.

• School-based digital interventions significantly reduce knowledge gaps.

• Greatest knowledge retention after 3 months was for alcohol abstinence, having a healthy diet and increasing physical activity.

• Knowledge retention trend shows a need for reinforcement to obtain sustained results

**Supplementary Information:**

The online version contains supplementary material available at 10.1186/s12889-026-26442-0.

## Background

Cancer is among the most prevalent diseases in Germany, with approximately half a million new diagnoses annually [1]. In fact, cancer is the second leading cause of death in Germany after cardiovascular diseases, and among the leading causes of death worldwide [2]. Epidemiological evidence estimates that approximately 40% of cancer could potentially be prevented by avoiding modifiable risk factors [3–6].

A public health initiative was established and coordinated by the International Agency for Research on Cancer (IARC), which developed the European Commission European Code Against Cancer (ECAC)[7]. The aim of the ECAC was to systematically develop 12 evidence-based actions that fall within the individual locus of control to reduce cancer risks. When these recommendations are correctly implemented, they can significantly reduce cancer incidence [7]. However, an increasing body of evidence demonstrates that with regards to cancer prevention a knowledge gap exists regarding what lifestyle changes may be appropriate to prevent cancer [8, 9].

Adolescence represents a critical period for the formation of health-related attitudes and behaviours [10, 11]. It is known that habits which are formed in adolescence persist into adulthood and are difficult to modify later in life [12, 13]. Although not always translated into behavioural changes, increasing knowledge is an essential step to adopting behaviour changes [14]. Empowering adolescents to integrate habits that are proven to prevent cancer, can potentially prevent cancer incidence and mortality in decades to come, making this population an ideal subgroup in which to address existing knowledge gaps [15].

Evidence shows that initiatives aiming to increase knowledge regarding cancer risk reduction among school-aged adolescents could potentially have a lasting impact on public health outcomes [16, 17]. Simultaneously, an increasing body of evidence suggests that the mode of instruction, whether digital or in-person, does not significantly impact learning outcomes [18, 19]. Since digital and interactive formats are well-received by adolescents [20], the increasing digitization of schools offers new opportunities to implement health education projects in digital formats while overcoming the geographical limitations.

To date, there is limited research that addresses knowledge gaps regarding the 12 ECAC recommendations among adolescents and the extent to which digitally based interventions can effectively convey the principles of the ECAC to adolescents remains unclear. Therefore, this study aims to address this research gap by evaluating the effectiveness of a multimedia enhanced educational intervention grounded in the ECAC (version 4) recommendations [7]. Specifically, we aim to assess adolescents’ baseline knowledge related to cancer prevention, and to determine whether targeted multimedia instruction can begin to close knowledge gaps regarding the 12 ECAC recommendations.

## Methods

This prospective interventional study was conducted among secondary school adolescents in Bavaria, Germany attending 8th to 12th grades (aged approximately 13–19 years) from all tiers of the secondary school system. All data was collected in the framework of a publicly funded controlled study between November 2022 to April 2025 (Funding code: K1-2497-GLB-21-V7). Pre- and post-intervention anonymous, single point questionnaires assessed baseline knowledge and knowledge gains post-intervention. The primary endpoint was defined as a statistically significant average increase in knowledge operationalized as a minimum average improvement of 2-point improvement on the 12 ECAC recommendations immediately after the intervention and at the three months follow up. The present manuscript focuses on baseline data, primary outcomes and highlights.

### Development of the intervention

Analogous to the multifaceted collaborative approach described by Lawrence et al.[8], our multimedia-enhanced intervention utilized diverse teaching methods developed within an interdisciplinary team. Thus, we could incorporate expertise from diverse health professionals such as physicians, scientists, psycho-oncologists, and pedagogical specialists. All material was based on both the ECAC and the guidelines of the Bavarian State Ministry of Education and Cultural Affairs enabling a seamless integration of the material into the pupils’ general education plan [7, 21].

Foundational knowledge was presented in short (10–20 min) targeted animated videos. The short video sequences were followed by hands on tasks designed to stimulate critical thinking, foster discussion, and peer interaction. Such tasks included facilitated classroom discussions, mathematical problems related to the content (e.g. individual protein requirements, blood alcohol levels), analysis of medical pictures and diagrams (e.g. identifying a tumor on an X-ray image), and short, digital multiple-choice quizzes (e.g. “As of age can you get the human papilloma virus (HPV) vaccination?”) The lessons also included motivational factors developed by the psycho-oncologists specializing in adolescence and culminated in a live-online round table question-and-answer session with carefully selected experts from the fields of medicine (e.g., oncology, dermatology, radiation etc.) as well as sport and nutrition scientists, health literacy experts, and psycho-oncologists.

Psycho-oncologists, including those with a background in child and adolescent psychotherapy, where involved in all aspects of the intervention. They not only provided material to the onsite school psychologists but were available throughout the intervention for any immediate support needs among participants. They additionally contributed to the round table addressing any fears expressed and/or questions the pupils may have had regarding the psycho-sociological effects of cancer. Recognizing that addressing the topic of cancer may elicit emotional discomfort and distress, psycho-oncologists also played an essential role in the development of age-appropriate educational content. For example, the psycho-oncologist helped to intentionally design our intervention to carefully balance the well-established scientific evidence linking obesity to cancer risk with potential psychological risks associated with emphasizing body weight during adolescence. Their involvement ensured that all material was sensitively tailored to support adolescents, enabling them to engage with the subject constructively.

The finalized lessons, including the animated videos and all material underwent pilot testing among teachers (*n* = 3), educators (*n* = 2), experts (*n* = 8), and adolescents (*n* = 8), utilizing a step-by-step Delphi-oriented feedback process aimed at refining content and delivery. A facilitator then summarized the feedback and enabled participants to reconsider and refine their suggestions. The process continued until a majority consensus was achieved on key aspects of the lesson plans. This approach ensured that the final content reflected the collective interdisciplinary expertise and as well as a representative of end-users.

### Implementation of the Intervention

The intervention was spread over six consecutive 45-min lessons delivered over the course of a single day and led by the classroom teachers. During the last 45-min session, the pupils’ questions were addressed in a live-online round table consisting of experts from the fields of medical oncology, nutrition, exercise, psycho-oncology and health literacy. The teachers were briefed thoroughly before the intervention and received a detailed lesson plan outlining the timeline and their role and including answer keys. The teachers were then responsible for supervising the pupils throughout the intervention and providing the questions the pupils came up with anonymously to the expert team during the live-online round table.

### Recruitment

A multi-stage recruitment strategy, which combined random sampling of schools with mixed-mode outreach methods, was used to ensure that a diverse population scattered through Bavaria was included. To this end, a sample of potential schools were randomly selected from a government issued list of schools spanning all seven government districts. In each district, 80 schools were then contacted by email and invited to participate. Additionally, the program was advertised in different public forums, including newsletters issued for teachers, newspapers, and a program website,^1^ as well as through word of mouth. The interested schools were then responsible for identifying a subset of pupils as specified in our inclusion criteria.

### Inclusion criteria

All school types in the Bavarian system were eligible to participate. Inclusion criteria were defined as all pupils attending participating secondary schools with a working internet connection and necessary equipment (e.g. Beamer, microphones, internet enabled device approved for classroom use). Pupils who were in grades 5–13 were eligible to participate. Each provided informed consent. Accommodations were established for any pupil who chose not to participate.

### Study questionnaire

**Fig. 1:**
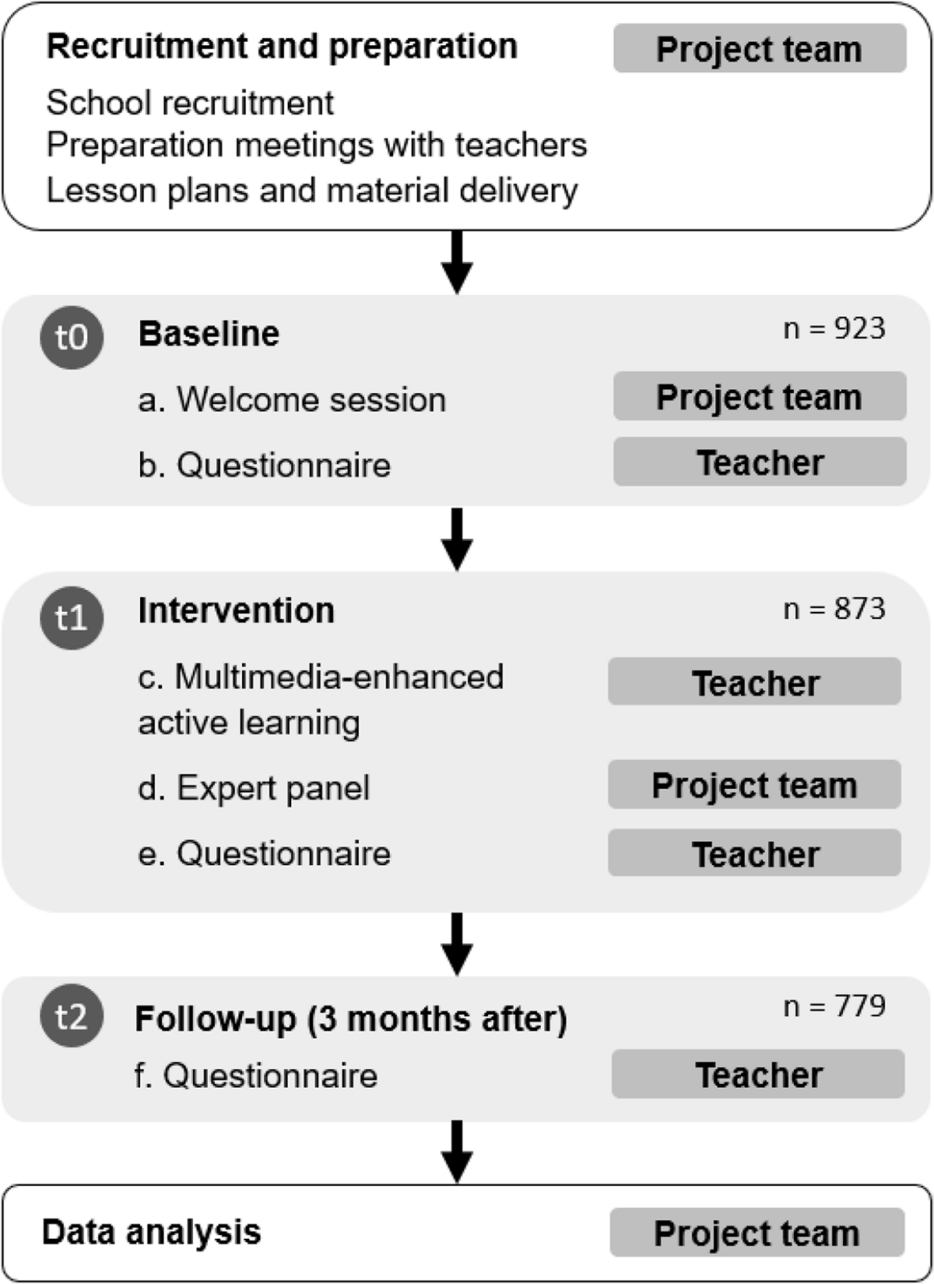
Study flow chart. Intervention process with responsibility and timing of the questionnaire. Abbreviations: t0 (baseline); t1 (post intervention), and t2 (three month follow up); Q (Questionnaire)

The questionnaire was designed to systematically assess basic knowledge of the 12 risk factors for preventing cancer outlined in the ECAC. It was delivered anonymously in paper form and repeated at all three time points: Immediately preceding the intervention (baseline) (t0), immediately post intervention (t1), and at three months follow up (t2) (Fig. 1). It consisted of the following single-recall-based, open-ended item: “The European Cancer Code lists 12 ways to reduce cancer risk. How many of the recommendations can you list?” The open-ended format was carefully chosen to improve accuracy and avoid overestimating knowledge gains [22]. To maintain assessment integrity, supervising classroom teachers were directed to administer the questionnaire in a controlled environment, similar to an examination protocol, thereby ensuring that all participants completed the questionnaire independently and without assistance. (Questionnaire can be found in Suppl. Q1). Teachers were additionally instructed to only include the pupils who signed the informed consent and were present for the intervention when completing the follow up questionnaire after three months (t2).

### Data analysis

Prior to commencing the intervention, a power analysis was conducted to determine the required sample size. Based on the effect sizes reported in the study by Davies et al. [23], and assuming an alpha level of 0.05 and a power of 80%, the target sample size was set at n = 552. This calculation was performed using PS Power and Sample Size [24]. Quantitative variables were derived by converting categorially demographic data, such as grade level, school type, and gender into numerical codes. School level was classified as lower (grades 8–10) or upper (grades 11–13). Categorical responses to open-ended questions were classified as “correctly recalled”, “partially recalled,” or “not recalled”. Responses were scored numerically as follows: 1 point for correct identification, 0.5 points for partial identification, and 0 points for no identification. Descriptive statistics were computed, with quantitative variables summarized by mean and standard deviation, and qualitative variables by absolute and relative frequencies. As state regulations mandated anonymous data collection, it was not possible to calculate individual-level changes. Therefore, ANOVA was used for the analysis of knowledge scores across the time points, and Tukey’s post-hoc test was applied to adjust for multiple comparisons across all pairwise mean differences. A binomial test was calculated to test the significance of the increase in the proportion of knowledge between the three points in time. To assess the influence of demographic and educational variables (gender, school type, and school level), linear regression models were employed. School types which typically lead to vocational education were aggregated into a single category “vocational track” and compared to the “academic track”. To evaluate the sustainability of knowledge retention, a sustainability quotient was calculated: (t2-t0)/(t1-t0). This was used to calculate the percentage increase in knowledge comparing the difference between the follow-up and the previous knowledge (t2-t0) with the direct increase in knowledge as a result of the intervention (t1-t0). A value close 1 thus indicated full retention of the respective recommendations.

### Data management and privacy

All data handling and storage procedures were in strict accordance with the General Data Protection Regulation (DSGVO). Access to anonymized data was restricted to authorized personnel. Schools were assigned unique codes to ensure anonymity while allowing for institutional-level analyses. The expert panel joined remotely and cameras from the participating schools were switched off.

### Ethical and legal considerations

The study was conducted in accordance with the Declaration of Helsinki and was approved by the Ethics Committee of the Medical Faculty of the Ludwig-Maximilian-University Munich (Nr. 21–0454). Additionally, permission to conduct the study during school hours was obtained from the Bavarian State Ministry of Education and Cultural Affairs.

Informed consent was obtained from the pupils themselves (if over the age of 16) or their legal caregivers. It was indicated that participation in the study is voluntary and all data would be collected anonymously.

## Results

### Participants

A total of *n* = 923 pupils from 14 schools consented and participated in the baseline assessment (t0) (Table 1). Follow-up assessments included *n* = 873 pupils at t1 and *n* = 779 at t2. At t0, the study population exhibited a gender distribution comprising of *n* = 478 female participants (51.8%), *n* = 360 males (39.0%) and *n* = 25 (2.7%) identifying as diverse. Notably, a small proportion of respondents (*n* = 60, 6.5%) did not specify their gender, reflecting a typical pattern of non-response for sensitive demographic queries in school-based research [25, 26]. The majority of participants were enrolled in the lower secondary level (grades 8–9), accounting for 694 pupils (75.2%), while 229 pupils (24.8%) were classified as upper secondary (grades 10–12). The sample was characterized by a slight predominance of pupils in the academic track (*n* = 552, 56.6%). The German academic track provides a diploma enabling direct and unrestricted entry into any university. The remaining *n* = 401 (43.4%) of the sample was made up of pupils from the vocational track. The Vocational track diploma is more practical, career-oriented route which enables subject-restricted university access.

**Table 1:** Sociodemographic characteristics

	t0 (***n*** = 923)		t1 (***n*** = 873)		t2 (***n*** = 779)	
	N	%	N	%	N	%
Gender						
Female	478	51.79	456	52.23	400	51.35
Male	360	39.00	307	35.17	297	38.13
Diverse	25	2.71	37	4.24	27	3.47
NA	60	6.50	73	8.36	55	7.06
School track						
Academic track	522	56.55	501	57.39	484	62.13
Vocational upper secondary	209	22.64	178	20.39	110	14.12
Vocational track	192	20.80	192	21.99	185	23.75
NA	0	0.0	2	0.23	0	0.0
School level						
Lower secondary level	694	75.19	673	77.09	655	84.08
Upper secondary level	229	24.81	189	21.65	124	15.92
NA	0	0.0	11	1.26	0	0.0

Legend: t0 (baseline); t1 (post intervention) and t2 (three month follow up); NA (not available). Academic track (German: Gymnasium); Vocational upper secondary school (German: Fachoberschule & Berufsoberschule); Vocational track (German: Mittelschule&Realschule). Lower Secondary: grades 8–10: Upper Secondary level: grades 11–13

### Primary endpoint

**Fig. 2:**
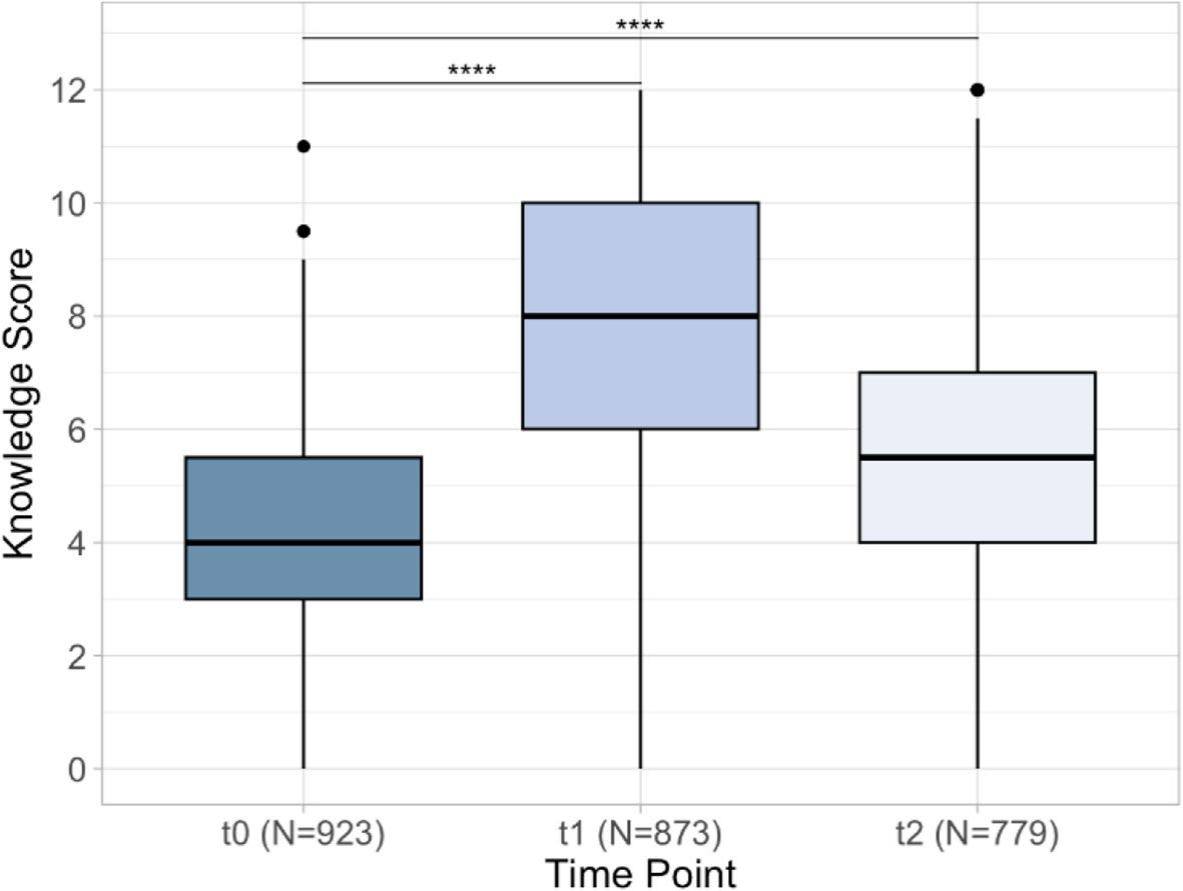
Knowledge scores over three time points. Legend: t0 (baseline); t1 (post intervention) and t2 (three month follow up)

A significant difference was observed among our population in mean knowledge scores across all three assessment time points (*p* < 0.0001) (Fig. 2). Specifically, post-intervention knowledge at t1 (M = 7.63 (SD = 2.67)) was significantly higher than at baseline (t0, M = 4.11 (SD = 1.84), *p* < 0.001). Knowledge also remained significantly elevated at the three-month follow-up when compared to baseline (t2, M = 5.70 (SD = 2.45), *p* < 0.001). Relative to baseline a trend toward knowledge loss at t2 was observed, however, this decline did not reach the threshold of statistical significance (*p* > 0.05).

### Gender differences in knowledge acquisition

Significant increases in knowledge scores were observed from baseline (t0) to post-intervention (t1) and at three-month follow-up (t2) for both males (*n* = 360) and females (*n* = 478) (*p* < 0.001) (Supplementary Fig. 1). Compared to their male counterparts’ female participants consistently exhibited significantly higher knowledge scores regarding the recommendations across all assessment time points (*p* < 0.001). Specifically, at baseline, female pupils had, on average, knowledge scores that were 0.78 points higher than those of male pupils (*p* < 0.001) (Supplementary Table 1.). Participants who identified as gender diverse (*n* = 25) also demonstrated a significant increase in knowledge between baseline (t0, MD = 4.50 [2.00, 8.00]) and post-intervention (t1, MD = 7.50 [3.00, 12.0]), with a gain of 3 points following the intervention (t2, MD = 4.50 [2.00, 9.50]); *p* < 0.001). These findings demonstrate a significant and sustained improvement in knowledge following the intervention.

### Knowledge scores according to recommendation

Table 2 presents a detailed overview of knowledge scores for all 12 recommendations specified by the ECAC at all three time points, as well as an overview of the sustainability of knowledge retention for each recommendation over time. It is notable that, at baseline (t0), multiple knowledge gaps of the ECAC recommendations were apparent. In fact, at baseline, participants showed the lowest levels of knowledge concerning recommendations related to breastfeeding (1.2%), second-hand smoking (2.0%), and maintaining a healthy body weight (4.9%). Conversely, the most frequently named recommendations at baseline were abstaining from smoking (85.4%), maintaining a healthy dietary pattern (68.9%), and engage in regular physical activity (55.1%). When compared to baseline knowledge (t0), the greatest total knowledge increase immediately after the intervention (t1-t0) was observed for the recommendation to breastfeed (+ 50%; 1.20% (t0), 51.2% (t1), *p* < 0.001). This was followed by an increase in knowledge pertaining to participation vaccination programs (+ 49.6%; 13.9% (t0), 63.5% (t1), *p* < 0.001) and regular physical activity (+ 39.4%, 55.1% (t0), 94.5% (t1), *p* < 0.001). At three months follow up (t2) the lowest general knowledge persisted for following recommendations: second-hand smoking (16.0%, with an increase (t2-t0) of 14.0%), healthy body weight (17.1%, with an increase (t2-t0) of 12.2%), and cancer-causing substances (17.2%, with an increase (t2-t0) of 2.7%). Importantly abstinence from alcohol was the most sustainably recalled recommendation [calculated by t2-t0)/(t1-t0)] recalled by pupils (0.76). Healthy dietary pattern was the second most sustainably recalled recommendation (0.72). Physical activity ranked third in terms of sustained knowledge retention (0.69).

**Table 2:** Prevalence and changes in knowledge of risk factors

ECAC-recommendation % (n)	t0 (*n* = 923)	t1 (*n* = 873)	t2 (*n* = 779)	Change t1-t0	*p*-Value	Change t2-t1	*p*-Value	Change t2-t0	*p*-Value	Sustain-ability (t2-t0)/(t1-t0)
Abstain from smoking	85.4 (788)	91.1 (795)	87.8 (684)	5.7	< 0.001	−3.3	0.001	2.4	0.029	0.42
Avoid second hand smoke	2.0 (18)	33.6 (293)	16.0 (125)	31.6	< 0.001	−17.6	< 0.001	14.0	< 0.001	0.44
Maintain a healthy body weight	4.9 (45)	40.5 (354)	17.1 (133)	35.6	< 0.001	−23.4	< 0.001	12.2	< 0.001	0.34
Regular Physical Activity	55.1 (509)	94.5 (825)	82.3 (641)	39.4	< 0.001	−12.2	< 0.001	27.2	< 0.001	0.69
Follow a healthy dietary pattern	68.9 (636)	94.6 (826)	87.3 (680)	25.7	< 0.001	−7.3	< 0.001	18.4	< 0.001	0.72
Alcohol abstinence	45.9 (424)	83.4 (728)	74.5 (580)	37.5	< 0.001	−8.9	< 0.001	28.6	< 0.001	0.76
Protect from sun exposure	30.6 (282)	67.6 (590)	51.9 (404)	37.0	< 0.001	−15.7	< 0.001	21.3	< 0.001	0.58
Protect from cancer-causing substances	14.5 (134)	31.3 (273)	17.2 (134)	16.8	< 0.001	−14.1	< 0.001	2.7	0.021	0.16
Avoid radiation exposure	12.4 (114)	36.9 (322)	23.4 (182)	24.5	< 0.001	−13.5	< 0.001	11.0	< 0.001	0.45
Importance of breastfeeding	1.20 (11)	51.2 (447)	21.4 (167)	50.0	< 0.001	−29.8	< 0.001	20.2	< 0.001	0.40
Participate in vaccination programs	13.9 (128)	63.5 (554)	34.0 (265)	49.6	< 0.001	−29.5	< 0.001	20.1	< 0.001	0.41
Early detection screening awareness	32.0 (295)	55.3 (483)	35.0 (273)	23.3	< 0.001	−20.3	< 0.001	3.0	0.036	0.13

Legend: Prevalence and changes in knowledge of risk factors across three time points, including sustainability of information retention. ECAC (European Code Against Cancer); t0, (baseline); t1 (post intervention) and t2 (three month follow up)

### Comparison of school type and grade level

Significant improvements in knowledge scores were also observed within each school type and sustained at three-month follow-up (*p* < 0.001) (Supplementary Fig. 2). Similarly, both lower and upper secondary level pupils from both tracks showed significant increases from baseline to post-intervention and after the three-month follow-up (*p* < 0.001) (Supplementary Fig. 3). At baseline, upper-level secondary pupils had significantly higher knowledge when compared to lower-level secondary pupils (MD = 1.23, *p* < 0.001). While this difference attenuated post-intervention, it was again significantly higher when compared to baseline at follow-up (*p* = 0.0007).

## Discussion

To our knowledge, this is the first study that combines the recommendations of the ECAC with a digital and interactive program for adolescents in a school setting [8]. Our findings demonstrate that the intervention resulted in an immediate effect, with a sustained, albeit attenuated impact at three months post-intervention. In fact, we were able to demonstrate significant knowledge increases of the recommendations across the entire study population. It should be noted that these results also show a trend towards lower retention at the three-month follow-up when compared to baseline. These results align with recent studies indicating that awareness of evidence-based guidelines, such as the ECAC, remains limited within the general population and reflect the need for such targeted interventions [8, 9, 27, 28]. In fact, every piece of knowledge retained enables individuals to recognize risk factors and sets the foundation for early adoption of protective behaviours, which is critical for reducing future cancer incidence [15].

Nonetheless, our results reflect a non-significant decline in knowledge between t1 and t2, indicating that knowledge gains may be limited or not fully sustained. This pattern is consistent with the findings of Hubbard et al. who also reported a modest decrease in knowledge six months following the intervention [29]. Similarly, Cameron et al. report a decrease in knowledge of around 10% above the initial level 4 months after the intervention [30]. To address this decline, they advocated for the integration of supplementary strategies, such as mobile applications and gamification, to reinforce learning and retention. Furthermore, Lawrence et al. highlight the importance of frequent and repeated interventions to maintain knowledge gains [8]. Our results confirm the importance of future repetition within public health programs and integration of supplementary strategies in order to achieve lasting improvements in knowledge, and ultimately public health gains.

Abstinence from alcohol was the most sustainably recalled recommendation across all time points, likely reflecting strong public awareness of alcohol’s health risks despite its social acceptance and industry influence. This suggests that while knowledge about alcohol harms is robust, stricter regulations in Germany may be needed to foster behavioural change [31]. Healthy nutrition ranked second, possibly due to the daily relevance of food choices that reinforce knowledge retention. Although physical activity was highly recognized at baseline, it ranked third after three months, indicating sustained knowledge gains. The lasting recall of these lifestyle-related recommendations highlights the intervention’s effectiveness in linking cancer prevention guidance to everyday adolescent life.

In contrast, second-hand smoking was among the least frequently recalled preventive recommendations at all three time points in our study. This contrasts with findings from a study conducted among Australian adolescents, in which 70% named second-hand smoke as a major risk factor [32]. In fact, it was ranked as the second most frequently recalled recommendation after smoking. Similarly, Di Giuseppe et al. reported that 63.8% of Italian adolescents recognized second-hand smoking as a cancer risk factor [17]. Another study in the United Kingdom demonstrated that approximately 60% of adolescents considered second-hand smoke to be a risk factor. The contrast could be explained by the fact that anti-smoking campaigns in Germany focus on active smoking risks, while the messaging around second-hand smoking risks are inconsistent and less in realm of an adolescents’ locus of control [33].

Weight-related factors were among the least frequently recalled cancer risk factors in our study. This may be attributed to the intentional design of our intervention, in which we chose an indirect approach that prioritized the promotion of healthy dietary patterns and regular physical activity instead of focusing explicitly on body weight. Adolescence is a particularly critical developmental period during which individuals are vulnerable to body image concerns and the onset of eating disorders such as anorexia nervosa and bulimia [34]. Numerous studies have shown that overemphasis on body weight, in particular when tied to health risks such as cancer, can increase body dissatisfaction, anxiety, and unhealthy behaviour patterns which are recognized as precursors of eating disorders [34–37]. Furthermore, public health messaging focusing on body weight may unintentionally stigmatize certain body sizes or types, contributing to psychological distress and disordered eating patterns in youth [36, 38]. Our strategy thus allowed us to address body weight as a risk factor in a more supportive and non-stigmatizing manner, without excluding it entirely from the intervention.

Interestingly, while our intervention led to notable knowledge increases among adolescent participants when compared to their baseline knowledge for many recommendations, some results were unexpectedly high. In fact, knowledge about breastfeeding showed the largest increase at 50%. This aligns with evidence derived from the fields of psychology and neuroscience, which confirm that surprising information is more memorable [6, 39, 40]. The recommendation to reduce cancer risk through vaccinations yielded the second-largest increase. Similarly, Hubbard et al. identified a knowledge gain with respect to HPV vaccines [29]. Consistent with Kyle et al. physical inactivity as a cancer risk factor showed the third largest increase despite the high baseline knowledge, whereby the percent increases of 39.4% in our study were considerably higher [27].

Importantly, although the HPV vaccination is a highly effective and preventive measure against certain types of cancer, only 13.9% of pupils were aware of this at baseline. These findings contrast with McDonald et al. and Fleary et al. who respectively found that 27% and 31% of adolescents recognized HPV infection as a cancer risk factor [32, 41]. The low recognition of the HPV vaccines in our findings could be attributed to regionally low vaccination coverage in Germany and in Bavaria. In fact, in 2021, only approximately 54.6% of 15-year-old girls and 27% of boys nationwide were fully vaccinated against HPV, with Bavaria consistently reporting below-average coverage [42]. Research further suggests that HPV knowledge among adolescents in Germany is generally low, influenced by sociocultural norms, limited health education, and insufficient provider communication [43]. Despite these limitations our intervention was still able to demonstrate a 20.1% increase in the awareness of the effectiveness of vaccination programs.

Female pupils started with a higher baseline knowledge and maintained this advantage across all three measurement points. These findings align with previous research, which also found that females demonstrated a higher level of health literacy in comparison to males [44]. Interestingly, Kyle et al. also observed a greater knowledge gain among female participants despite a lower baseline knowledge [27]. However, a similar study conducted among school-aged adolescents in Germany in 2014 reported no significant gender difference in cancer related knowledge [28]. As the number of pupils identifying as diverse were so low, no scientifically valid comparison could be analysed. In general, differences among gender are influenced by multiple contextual factors, including prior relevance and cultural background and should be interpreted with caution [32, 45].

Knowledge trajectories differed according to grade level. Similarly, to McDonald et al., upper secondary pupils demonstrated greater baseline knowledge about cancer risk factors [32]. In contrast, pupils in lower grade levels exhibited substantial learning gains from the intervention. In fact, immediately after the intervention (t1), pupils in lower grade levels identified only 0.5 fewer recommendations than their older peers, indicating a high intervention effectiveness. These findings support the potential for early-stage health education programs to mitigate disparities in health literacy, while the observed trend shows that such interventions need to be supplemented with further strategies and repeated exposure to the ECAC recommendation.

### Strengths and limitations

A central strength of our study lies in the fact that our primary endpoint relied on the use of open-ended recall. This format is highly sensitive to meaningful learning and, thus, more accurately reflects that the pupils have internalized and understood the presented content. Moreover, our methodology aligns with the recent findings by Lawrence et al. emphasizing that secondary schools are an ideal setting for cancer education programs [8]. In fact, the authors highlighted the value of multifaceted collaborative approaches such as ours. Furthermore, our multi-stage recruitment strategy expanded our ability to reach a broad and diverse sample spread across the state of Bavaria. Such strategies are commonly used in school-based research and particularly effective when aiming to recruit schools and their pupils as research participants [46, 47].

However, due to ethical and administrative restrictions, pseudonymized individual-level data could not be collected, limiting the analysis to group-level means and preventing a more detailed examination of individual learning trajectories. Furthermore, we had to rely on non-probability sampling, which, while practical, could have limited the results by introducing potential sampling bias and generalizability.

It could be considered a key limitation that our intervention emphasizes recognition and basic understanding of all 12 ECAC elements instead of intensive, skill-focused teaching about one or two key domains. As a result, pupil’s knowledge level about each recommendation remained at an introductory level and the study could not assess outcomes associated with a deeper mastery of specific concepts such as behavior change. However, early exposure across multiple domains is theorized to support later knowledge consolidation by providing foundational cognitive and attitudinal ‘anchors’ for future learning experiences [48, 49].

Additionally, variations in baseline knowledge integrated into the academic curriculums across the varying school types (e.g., health-focused tracks) may also have influenced outcomes. Despite these limitations, the study provides important evidence for the implementation of cancer prevention education in schools and supports calls for multi-faceted, stakeholder-driven approaches using diverse educational formats [8].

## Conclusion

This study, to the best of our knowledge, is the first to integrate the 12 ECAC recommendations into a multimedia school-based intervention targeted at adolescents. Our results demonstrate that such interventions carried out by an interdisciplinary team could effectively contribute to knowledge increases regarding knowledge of the ECAC recommendations, while simultaneously confirming that notable gaps exist among adolescents. These results underscore the critical need for early, targeted intervention to address baseline knowledge gaps. While knowledge gains were sustained over a three-month period, a decline was evident, suggesting the need for repeated or supplementary educational strategies. Further research is necessary to explore long-term behavioral outcomes resulting from knowledge gains, the optimal intervention frequency to ensure sustainable knowledge gain, and adapted approaches which may reach a larger population of underserved groups. The findings of this research will inform future strategies for integrating cancer prevention education into school curricula and contribute to the broader goal of reducing the burden of cancer through early, evidence-based interventions.

## Supplementary Information

Supplementary Material 1.

Supplementary Material 2.

Supplementary Material 3.

Supplementary Material 4.

Supplementary Material 5.

## Data Availability

The majority of data generated or analyzed during this study are included in this published article and its supplementary information files. The rest of the datasets are available from the corresponding author upon reasonable request.

## Abbreviations

ANOVA: Analysis of variance
ECAC: European code against cancer
HPV: Human papilloma virus
IARC: International agency for research on cancer
M: Mean
SD: Standard deviation
t0: Baseline
t1: Post intervention
t2: Three month follow up
Q: Questionnaire

## Footnotes

1 https://www.ccc-muenchen.de/forschung-projekte/projekte-des-ccc-munchen/ccc-macht-schule-sup-digital-sup/8690207a1a7e0f01
