# Supplementary figures and images for: From knowledge to action: strengthening cancer prevention knowledge in schools among adolescents in Germany
Source: BMC Public Health. 2026 Feb 3;26:722. doi: 10.1186/s12889-026-26442-0 (PMC12930987; doi:10.1186/s12889-026-26442-0)

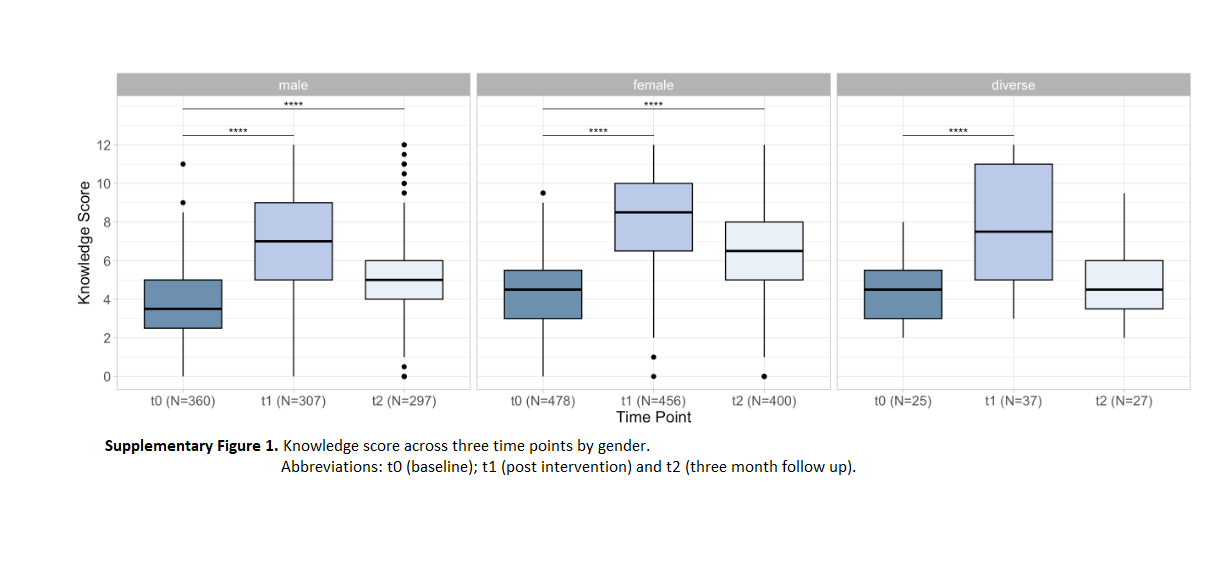

Supplement: Supplementary file 2 — Supplementary Material 2. [file 12889_2026_26442_MOESM2_ESM.png]

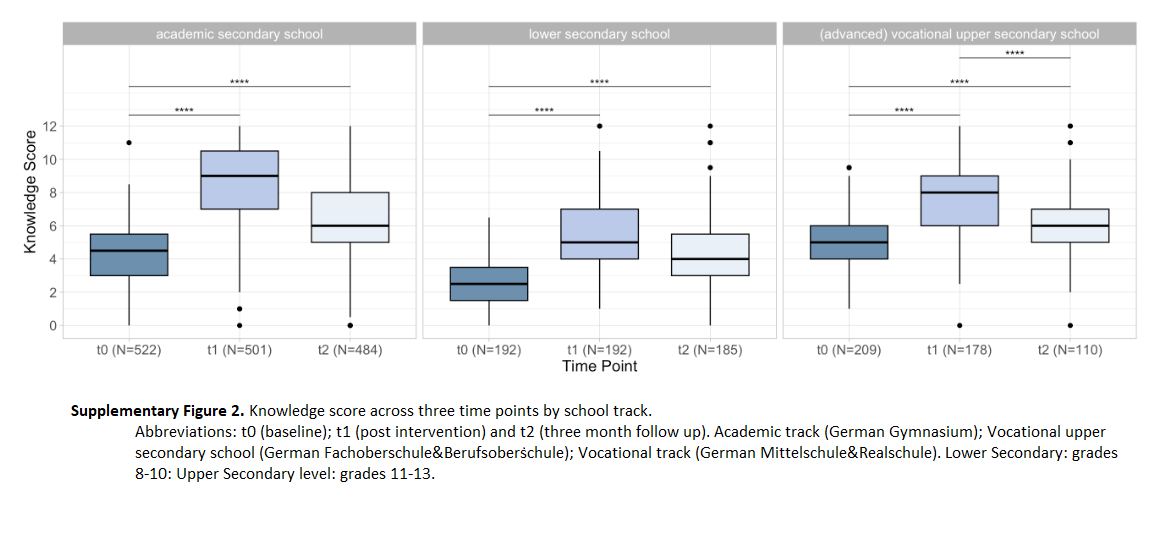

Supplement: Supplementary file 3 — Supplementary Material 3. [file 12889_2026_26442_MOESM3_ESM.png]

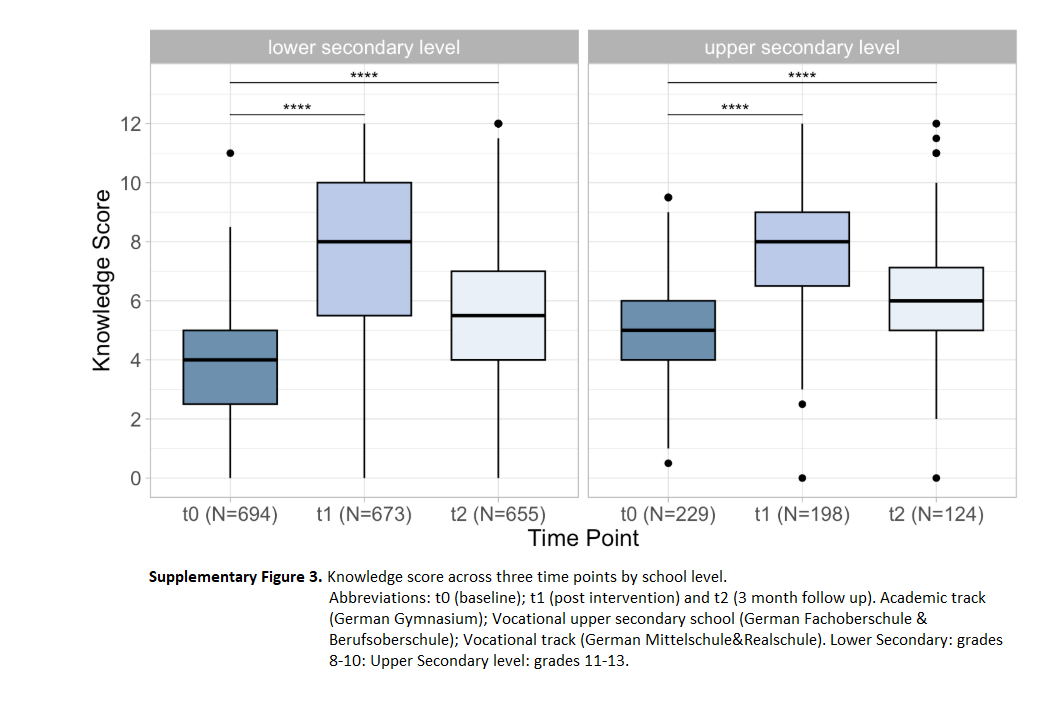

Supplement: Supplementary file 4 — Supplementary Material 4. [file 12889_2026_26442_MOESM4_ESM.png]
