# Supplementary material for: From knowledge to action: strengthening cancer prevention knowledge in schools among adolescents in Germany
Source: BMC Public Health. 2026 Feb 3;26:722. doi: 10.1186/s12889-026-26442-0 (PMC12930987; doi:10.1186/s12889-026-26442-0)
Supplement: Supplementary file 5 — Supplementary Material 5. [file 12889_2026_26442_MOESM5_ESM.docx]

**Supplementary Table 1.** Differences in Categories.

| **Time** | **Variable** | **Effect** | **p-Value** |
| --- | --- | --- | --- |
| t0 | female vs. male | 0.78 (0.53, 1.03) | <0.0001 |
| t0 | diverse vs. male | 0.79 (0.06, 1.53) | 0.0344 |
| t1 | female vs. male | 1.31 (0.94, 1.68) | <0.0001 |
| t1 | diverse vs. male | 1.06 (0.19, 1.93) | 0.0174 |
| t2 | female vs. male | 1.36 (1.01, 1.71) | <0.0001 |
| t2 | diverse vs. male | 0.03 (-0.9, 0.95) | 0.9571 |
|  |  |  |  |
| t0 | upper vs. lower secondary level | 1.23 (0.96, 1.49) | <0.0001 |
| t1 | upper vs. lower secondary level | 0.23 (-0.19, 0.66) | 0.2816 |
| t2 | upper vs. lower secondary level | 0.81 (0.34, 1.27) | 0.0007 |
|  |  |  |  |
| t0 | lower secondary vs. academic secondary school | -1.88 (-2.15, -1.62) | <0.0001 |
| t0 | vocational upper vs. academic secondary school | 0.84 (0.59, 1.1) | <0.0001 |
| t1 | lower secondary vs. academic secondary school | -3.27 (-3.66, -2.88) | <0.0001 |
| t1 | vocational upper vs. academic secondary school | -0.77 (-1.16, -0.37) | 0.0002 |
| t2 | lower secondary vs. academic secondary school | -1.91 (-2.3, -1.52) | <0.0001 |
| t2 | vocational upper vs. academic secondary school | 0.06 (-0.41, 0.54) | 0.7934 |

Abbreviations: t0, (baseline); t1 (post intervention), and t2, (three month follow up). Academic track (German Gymnasium); Vocational upper secondary school (German Fachoberschule&Berufsoberschule); Vocational track (German Mittelschule&Realschule). Lower Secondary: grades 8-10: Upper Secondary level: grades 11-13.
