# Supplementary material for: From knowledge to action: strengthening cancer prevention knowledge in schools among adolescents in Germany
Source: BMC Public Health. 2026 Feb 3;26:722. doi: 10.1186/s12889-026-26442-0 (PMC12930987; doi:10.1186/s12889-026-26442-0)
Supplement: Supplementary file 1 — Supplementary Material 1. [file 12889_2026_26442_MOESM1_ESM.pdf]

CCC MÜNCHEN
COMPREHENSIVE
CANCER CENTER
macht Schule

Open-ended Questionnaire

Gender: O male O female O diverse

<u>Knowledge: 12 Ways to reduce cancer</u>

In order to maintain anonymity, we request that you <u>DO NOT</u> provide any information on this questionnaire which would enable us to identify you personally (e.g.name or date of birth).

The European Cancer Code lists 12 ways to reduce cancer risk. How many of the recommendations can you list?
